# Associations between HIV and Human Pathways Revealed by Protein-Protein Interactions and Correlated Gene Expression Profiles

**DOI:** 10.1371/journal.pone.0034240

**Published:** 2012-03-27

**Authors:** Kuang-Chi Chen, Tse-Yi Wang, Chen-hsiung Chan

**Affiliations:** 1 Department of Medical Informatics, Tzu Chi University, Hualien, Taiwan; 2 Institute for Information Science, Academia Sinica, Taipei, Taiwan; University of South Florida College of Medicine, United States of America

## Abstract

**Background:**

AIDS is one of the most devastating diseases in human history. Decades of studies have revealed host factors required for HIV infection, indicating that HIV exploits host processes for its own purposes. HIV infection leads to AIDS as well as various comorbidities. The associations between HIV and human pathways and diseases may reveal non-obvious relationships between HIV and non-HIV-defining diseases.

**Principal Findings:**

Human biological pathways were evaluated and statistically compared against the presence of HIV host factor related genes. All of the obtained scores comparing HIV targeted genes and biological pathways were ranked. Different rank results based on overlapping genes, recovered virus-host interactions, co-expressed genes, and common interactions in human protein-protein interaction networks were obtained. Correlations between rankings suggested that these measures yielded diverse rankings. Rank combination of these ranks led to a final ranking of HIV-associated pathways, which revealed that HIV is associated with immune cell-related pathways and several cancer-related pathways. The proposed method is also applicable to the evaluation of associations between other pathogens and human pathways and diseases.

**Conclusions:**

Our results suggest that HIV infection shares common molecular mechanisms with certain signaling pathways and cancers. Interference in apoptosis pathways and the long-term suppression of immune system functions by HIV infection might contribute to tumorigenesis. Relationships between HIV infection and human pathways of disease may aid in the identification of common drug targets for viral infections and other diseases.

## Introduction

Acquired immunodeficiency syndrome (AIDS) is a devastating disease that has afflicted the human species for decades. Despite the enormous amount of effort and resources devoted to its study, a cure for AIDS has not yet emerged. AIDS is caused by human immunodeficiency virus (HIV). Similar to other diseases caused by pathogens, various human pathways must be perturbed or even hijacked to serve the purposes of the HIV virus. Indeed, hundreds of human host factors have been identified as necessary during viral infection and replication [1]–[3]. Thousands of protein-protein interactions between HIV and human host proteins have been reported in the literature [4].

Certain diseases are known to be associated with HIV infection. For example, the association between HIV/AIDS and lymphoma/Karposi's sarcoma has been recognized since the discovery of HIV [5]. Tuberculosis, hepatitis B/C, and other diseases are known comorbidities of HIV infection [6], [7], and HIV infection is even associated with neurocognitive disorders [8]. These findings have led us to enquire into the human pathways and diseases that are associated with AIDS and the molecular mechanisms behind these associations.

Previous research has attempted to elucidate host-pathogen interactions through protein-protein interactions. Interactions between human proteins and several pathogens, including Hepatitis C virus [9], Epstein-Barr virus [10], influenza virus [11], and several strains of bacteria [12], were identified systematically. These studies suggested that interactions between humans and pathogens (viruses or bacteria) are extensive and prevalent. Several studies have also attempted to identify human biological processes that are influenced or perturbed by viruses [13], [14]. These studies depicted human-pathogen interactions from a global perspective by pooling interactions with different pathogens and identifying common mechanisms playing important roles in viral and bacterial infections. One study specifically analyzed the interactions between HIV-1 and human proteins [15] and found that HIV targeted proteins that were not involved in human diseases listed in the Online Mendelian Inheritance in Man (OMIM).

To study the functional enrichment of genes (the association of genes with a specific function or pathway), gene set enrichment analysis (GSEA) and its derivatives are widely adopted [16], [17]. In GSEA, genes are ranked by their correlations with phenotypes and an enrichment score (ES) is calculated to estimate whether genes from a gene set are clustered in the extreme regions (the bottom or top) of the ranked list. Some studies have applied GSEA to network/pathway analysis as well. For example, proteins in a protein-protein interaction network can be ranked by their degrees or by other centrality scores [13]. Enrichment scores for pathways or other gene sets can be calculated based on the ranks and clusters of genes from these pathways. GSEA can also be applied to the evaluation of HIV/pathway associations, but genes must be ranked by their relatedness with HIV first. The selection of ranking criteria would impact the results of enrichment analysis.

In this work, we explored links between HIV infection and other human pathways of disease through several approaches: investigating the overlap of human genes involved in AIDS and other pathways, examining recovered human-HIV interactions in other pathways, studying co-expression profiles, and identifying common interaction partners in a human PPI network. All these approaches were undertaken with human genes associated with HIV and genes involved in pathways of disease. Two hundred twenty (220) human pathways involved in disease from the Kyoto Encyclopedia of Genes and Genomes (KEGG) were evaluated and statistically compared with HIV host factors. Many tests found significant associations between gene expression and HIV, and all test scores were transformed into ranks. Rank combination of these results led to a final ranking of HIV-associated pathways that provided insight into AIDS comorbidities, their underlying molecular mechanisms, and novel potential treatment strategies. Data fusion or the combination of multiple sources of information are techniques that have been applied to prioritize genes [18] or drug candidates [19]. However, the application of these concepts to pathways is less common. To the best of our knowledge, this is the first study to combine the rankings of pathways through different approaches.

## Results

### Consensus in HIV Host Factors

**Figure 1:**
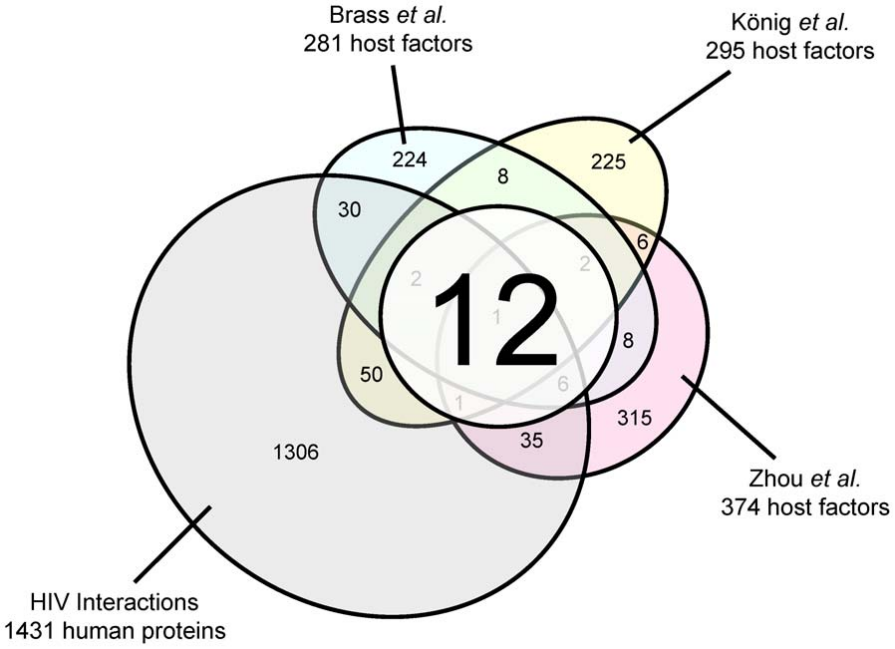
Venn diagram of HIV host factors. The numbers of common host factors reported in one, two, three or four sources are shown on respective cells. Only 12 host factors (white circle) were reported by more than three studies, and only one was reported in all four sources.

The HIV host factors identified among different studies are diverse. Figure 1 illustrates a Venn diagram of host factors identified from three systematic screening studies [1]–[3] and from HIV-human protein interactions reported in the literature [4]. Data from several sources can be merged with either set union or intersection operations. For the current study, the intersection approach was taken. As genes from our four sources were not balanced in terms of representation, the union of these data would make the results severely biased toward the largest set (HIV Interaction Database, 1,431 proteins). However, only one gene, RELA (a component of NF-κB), was consistently identified by all four sources. Therefore, genes identified by at least three sources were included for analysis, and twelve (12) host factors met this criterion (Table 1). These host factors were defined as a ‘core set’ for subsequent analysis in this work, and were referred to as ‘host factors.’ The degrees (numbers of interactions) of these genes in HIV-human and human-human protein-protein interactions and their respective ranks are also illustrated. Most of these host factors were not ranked highly. The human protein that interacted with the most HIV proteins was the gene product of MAPK1 (mitogen-activated protein kinase 1), whereas the human protein that interacted with the most human proteins was UBC (ubiquitin C). However, both proteins were not identified by the three systematic screenings as HIV host factors.

**Table 1:** Host factors identified in more than three studies.

Gene ID	Gene Symbol	Full Name	HIV Interactions	Brass et al.	Konig et al.	Zhou et al.	# of HIV Interactions	HIV Interaction Rank	# of Human Interactions	Human Interactome Rank
5970	RELA	v-rel reticuloendotheliosis viral oncogene homolog A (avian)	•	•	•	•	2	423/1431	155	36.5/11030
9972	NUP153	nucleoporin 153 kDa	•	•	•		1	980.5/1431	27	1031.5/11030
9443	MED7	mediator complex subunit 7		•	•	•	0	N/A	30	881.5/11030
920	CD4	CD4 molecule	•	•		•	8	6.5/1431	40	558.5/11030
9150	CTDP1	CTD (carboxy-terminal domain, RNA polymerase II, polypeptide A) phosphatase, subunit 1	•	•	•		1	980.5/1431	22	1362/11030
8534	CHST1	carbohydrate (keratan sulfate Gal-6) sulfotransferase 1	•		•	•	1	980.5/1431	1	9945.5/11030
7852	CXCR4	chemokine (C-X-C motif) receptor 4	•	•		•	4	113/1431	32	795.5/11030
6924	TCEB3	transcription elongation factor B (SIII), polypeptide 3 (110 kDa, elongin A)	•	•		•	1	980.5/1431	8	3749/11030
3716	JAK1	Janus kinase 1	•	•		•	1	980.5/1431	74	186/11030
207	AKT1	v-akt murine thymoma viral oncogene homolog 1	•	•		•	3	228.5/1431	156	35/11030
1654	DDX3X	DEAD (Asp-Glu-Ala-Asp) box polypeptide 3, X-linked	•	•		•	1	980.5/1431	23	1284.5/11030
10001	MED6	mediator complex subunit 6		•	•	•	0	N/A	30	881.5/11030

Previous analysis of protein-protein interactions between human proteins and various viruses has shown that many pathogenic viruses interact with ‘hubs’ (high degree nodes) in the human interaction network [13]–[15]. However, ranking host factors by their degrees did not reflect this property. Among the 12 host factors studied, only two (RELA and AKT1, ranked 36.5 and 35, respectively) were ranked within the top 100 of 11,030 human proteins with current interaction data available. As for HIV-human interactions, only CD4 was targeted by multiple HIV proteins, and CD4 was ranked 6.5 among 1,431 human proteins with HIV-human interaction data available.

### GO Annotation Enrichments of HIV Host Factors

**Figure 2:**
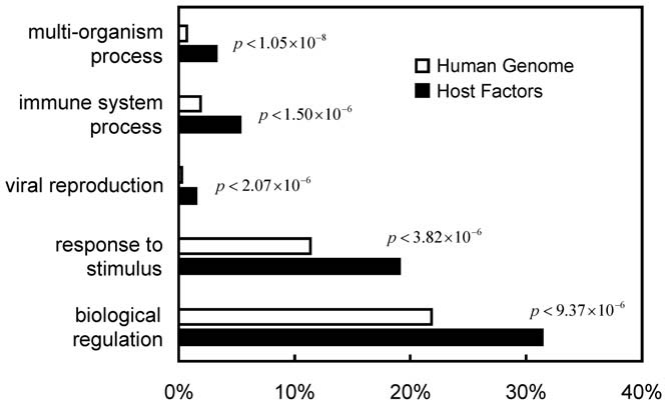
GO distribution. Significantly enriched GO terms between the human genome (empty bars) and HIV host factors (solid bars) are reported here. The *p*-values for these GO terms were all <1×10^−5^.

To understand the involvement of HIV host factors in biological processes, Gene Ontology (GO) annotations (biological processes) were compiled for host factors and compared to those of the entire human genome. For HIV host factors, ‘multi-organism process (GO:0051704)’, ‘immune system process (GO:0002376)’, ‘viral reproduction (GO:0016032)’, ‘response to stimulus (GO:0050896)’, and ‘biological regulation (GO:0065007)’ were significantly enriched (all with *p*-values<1×10^−5^, Figure 2). The definition of a ‘multi-organism process’ in Gene Ontology was: ‘Any process in which an organism has an effect on another organism of the same or different species (http://amigo.geneontology.org/cgi-bin/amigo/term_details?term=GO:0051704).’ Therefore, genes targeted by HIV are likely to be those involved in human-pathogen interactions. The enrichment of ‘immune system process’, ‘viral reproduction’ and ‘biological regulation’ is consistent with the behaviors of HIV and the consequences of HIV infection. The enrichment of ‘response to stimulus’ reflects the behaviors of cells in response to the binding or detection of the virus. These results are consistent with what is currently known about the virus, which includes its modulation of the immune system and its interference with cellular processes.

### Associations between HIV Host Factors and KEGG Pathways

There are 220 human pathways available in KEGG. Among these, 86 are metabolic pathways and the others belong to signaling pathways or pathways of disease. None of the metabolic pathways ranks in the top 10 by all four rankings (Supplementary Table S1). Almost all of the metabolic pathways are ranked in the bottom half of the list, with the overall pathway (hsa01100: Metabolic Pathway) ranked last. This suggests that HIV host factors are not greatly involved in metabolic processes, which is consistent with our GO enrichment/depletion analysis (Supplementary Table S2). The association between each pathway and a set of HIV host factors was evaluated using several approaches. Pathways were then ranked by statistical tests in comparison with random pathways. The nature of each approach led to different rankings for these pathways. Six pathways were ranked in the top 10 in at least three rankings. These consensus pathways include ‘Pancreatic cancer (hsa05212)’, ‘Small cell lung cancer (hsa05222)’, ‘Acute myeloid leukemia (hsa05221)’, ‘Adipocytokine signaling pathway (hsa04920)’, ‘B cell receptor signaling pathway (hsa04662)’, and ‘T cell receptor signaling pathway (hsa04660)’ (Supplementary Table S1).

**Figure 3:**
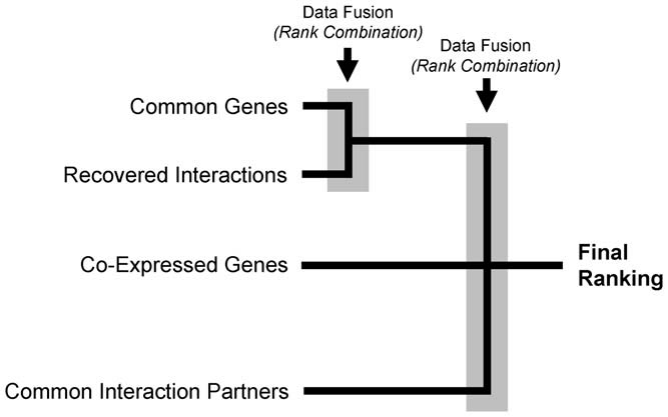
Rank combination scheme. Four measures were used in this study to rank KEGG pathways. Based on rank correlation, two (common genes and recovered HIV interactions) were merged by rank combination. The resulting three ranks were then combined again using the same data fusion technique.

To further explore the consensus pathways identified by the four approaches to analysis, a data fusion method was applied. The correlations among different rankings were calculated and are listed in Table 2. Two approaches were highly correlated, namely ‘Common Genes’ and ‘Recovered Interactions.’ The other correlations were less obvious, suggesting that these approaches yielded diverse results. In principle, rank combination of diversified results leads to better rankings [20], [21]. Based on these rank correlations, the ranks resulting from the four analytical approaches were combined as illustrated in Figure 3. The two most highly correlated rankings were combined first, as otherwise they would weigh too heavily when combined with the other rankings. The resulting three rankings were then combined again, resulting in the final ranking.

**Table 2:** Rank correlation coefficients among rankings of pathways identified by our four approaches.

	Common Genes	Recovered Interactions	Co-Expressed Genes	Common Interaction Partners
**Common Genes**				
**Recovered Interactions**	0.9933			
**Co-Expressed Genes**	0.5624	0.5576		
**Common Interaction Partners**	0.5432	0.5398	0.5822	

The top 10 KEGG diseases/pathways in the final ranking are listed in Table 3, along with their ranks and statistical significances as calculated by the four approaches. The six top-ranked consensus pathways were still ranked highly in the final ranking. However, four pathways were promoted by the combined ranking, namely ‘Chronic myeloid leukemia (hsa05220)’, ‘Toll-like receptor signaling pathway (hsa04620)’, ‘Chemokine signaling pathway (hsa04062)’, and ‘Apoptosis (hsa04210)’.

**Table 3:** Top 10 KEGG pathways by rank combination.

Combined Rank	Pathway Number	Pathway Title	Common Genes		Recovered Interactions		Co-Expressed Genes		Common Interaction Partners	
			Rank	*p*-value	Rank	*p*-value	Rank	*p*-value	Rank	*p*-value
1	05212	Pancreatic cancer	1	1.07×10^−15^	6	2.41×10^−12^	8	6.24×10^−5^	1	1.93×10^−14^
2	04660	T cell receptor signaling pathway	3	1.48×10^−10^	1	3.08×10^−27^	2	3.51×10^−10^	12	1.69×10^−9^
3	05221	Acute myeloid leukemia	4	3.11×10^−9^	3	9.04×10^−13^	16	7.37×10^−4^	2	3.53×10^−14^
4	04662	B cell receptor signaling pathway	9	7.69×10^−8^	8	4.36×10^−10^	11	1.61×10^−4^	5	3.92×10^−12^
5	05222	Small cell lung cancer	12	7.71×10^−7^	9	1.85×10^−9^	7	1.77×10^−5^	9	3.30×10^−10^
6	05220	Chronic myeloid leukemia	11	4.31×10^−7^	11	8.52×10^−8^	13	2.35×10^−4^	3	4.06×10^−14^
7	04920	Adipocytokine signaling pathway	5	4.44×10^−9^	5	2.21×10^−12^	23	1.45×10^−3^	4	2.72×10^−13^
8	04620	Toll-like receptor signaling pathway	16	8.07×10^−6^	13	1.11×10^−7^	12	1.93×10^−4^	13	2.08×10^−9^
9	04062	Chemokine signaling pathway	13	1.15×10^−6^	10	3.72×10^−8^	3	4.48×10^−8^	27	4.77×10^−6^
10	04210	Apoptosis	14	1.31×10^−6^	12	1.10×10^−7^	17	7.50×10^−4^	11	1.67×10^−9^

HIV particles must be granted entry into cells for successful infection and replication. It is thus understandable that ‘Chemokine signaling pathway’ was one of the top 10 pathways associated with HIV host factors. The glycoproteins gp160, gp120, and gp41 of HIV bind with CD4 and CXCR4/CCR5 on host cells before gaining entry into T cells. This binding triggers various signals throughout the cell, affecting the survival and migration of cells.

Three other pathways were involved in sensing and responding to viral infections, including ‘Toll-like receptor (TLR) signaling pathway’, ‘T-cell receptor (TCR) signaling pathway’, and ‘B-cell receptor (BCR) signaling pathway’. Activation of these pathways leads to immune responses including antigen processing and presentation, immunoglobulin production, and interferon-mediated antiviral effects. In some cases, activation of these pathways may also lead to autoimmunity.

Other gene expression-based studies also identified pathways associated with HIV infection [22], [23]. Our findings were consistent in identifying pathways identified in these studies, including ‘Apoptosis Pathway’, ‘Cytokine Responses’, and ‘Toll-like Receptor Pathway’ [22].

The cancers identified in this work were not HIV/AIDS-defining cancers and were not known to have been caused by infectious agents. However, various population-based studies have shown that the risks of contracting many of these cancers are elevated in people with HIV/AIDS. An epidemiological study in France showed that the incidence of acute myeloid leukemia (AML) in HIV/AIDS patients was two-fold higher than that of the general population [24]. One study in Germany suggested that long-term immune suppression increased AML risk [25]. The clinical evidence for associations between chronic myeloid leukemia (CML) and HIV/AIDS is less clear, though some studies have suggested that HIV infections and highly active anti-retroviral therapy (HAART) may increase the risk of CML [26]. Two studies in the United States and one in Denmark showed that the incidence of lung cancer increases in HIV-infected individuals [27] and that HIV infection is associated with an increased risk of lung cancer [28], [29]. Two studies in France [30] and Italy [31] also found that pancreatic cancer deaths were significantly higher in populations with HIV/AIDS.

The association between HIV and the ‘adipocytokine signaling pathway’ was less clear. However, HIV protease inhibitors and other anti-retroviral therapies have been shown to alter human adipocyte differentiation and metabolism [32], [33]. The underlying mechanism for this lipodystrophy might be due to mitochondrial toxicity and insulin resistance [34]. This association was noted in an RNAi systemic screening study [3].

## Discussion

Using a set of stringent and conserved host factors, it has been found that HIV does not always target ‘hubs’ or high-degree nodes in the human interactome. High-throughput screening of host-pathogen interactions may lead to interactions with already promiscuous proteins. Additionally, ‘hubs’ in a network are not necessarily involved in specific processes. Combining data from multiple sources reduced the number of false positives. Associations between a reliable ‘core set’ of HIV host factors and pathways or diseases may be more significant and specific, and reveal insights into the underlying molecular mechanisms of pathogenesis and comorbidities.

In conventional pathway enrichment methods (GSEA) all genes (host factors and genes in the human genome) must be ranked using a pre-specified criterion. Usually gene expression profiles of a certain phenotype (such as HIV infection) would be used. However, using this method, multiple factors or conditions cannot be considered together. Other than gene expression, the weight of evidence (number of independent studies reporting the gene being linked to the disease or condition) and degrees or centralities in protein-protein interaction networks could also be employed as ranking criteria. However, most of these criteria are unable to assign scores to all human genes, and would impact the calculations of enrichment scores and the ranking of pathways. Unlike the GSEA method, our method only requires a set of host factors. Associations between HIV and pathways are dependent on the set of HIV host factors. This is advantageous in terms of the computational complexity as the remaining genes in the human genome can be omitted from further study.

In this work, various cancer pathways were shown to be significantly associated with HIV. This observation is consistent with several studies investigating cancer risks in HIV/AIDS populations [27], [30], [31]. Why does HIV associate with diverse types of cancers? HIV is known to integrate its genetic materials into the host genome, which could be a cause of HIV-defining carcinomas. The random sites of integration of HIV might corrupt the expression of tumor-suppresser genes and alter the behaviors of cells. For other non-HIV-defining cancers, it is recognized that apoptosis (the killing of damaged cells) [35] and senescence (the inactivation of damaged cells) [36] play critical roles in tumorigenesis.

**Figure 4:**
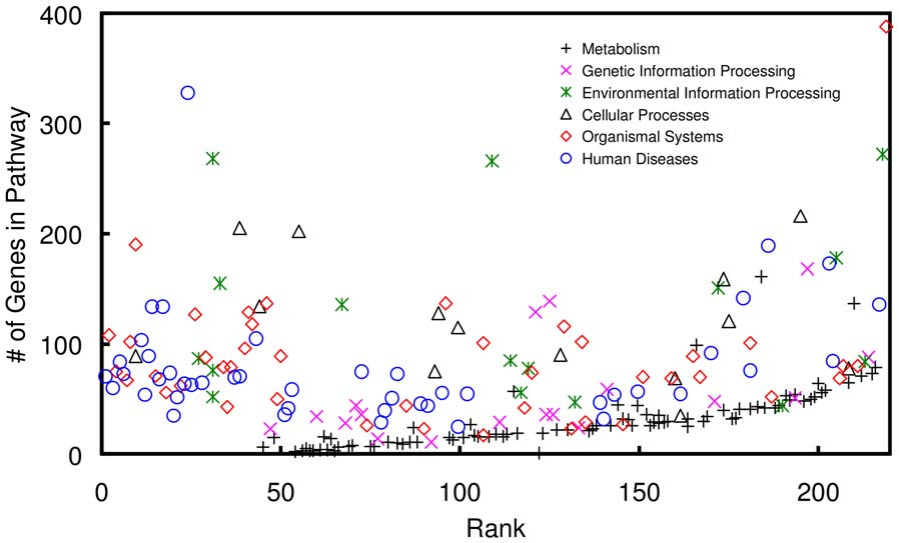
KEGG Pathway categories and ranks. The ranks of KEGG pathways are plotted against the numbers of genes in the pathways. Pathways are labeled according to their assigned categories on the KEGG website (http://www.genome.jp/kegg/pathway.html).

One concern over the associations revealed in this work is whether highly ranked pathways were simply those with more genes, as larger pathways may include more host factors by chance. The KEGG database contains various types of pathways, including ‘Metabolism’, ‘Genetic Information Processing’, ‘Environmental Information Processing’, ‘Cellular Processes’, ‘Organismal Systems’, and ‘Human Diseases’ [37]. Whether certain types of pathways would cluster at the top of the ranking may cause concern for the validity of the ranking results. To address these issues, the numbers of genes in pathways were plotted against the ranks of those pathways (Figure 4). The resulting figure illustrates that ranks are not correlated with the numbers of genes in pathways. Other than ‘Metabolism’, which tends to rank low, most pathways do not exhibit obvious trends of clustering.

Many of the host factors studied were significantly involved in the apoptosis pathway, notably AKT1 and RELA (part of NF-κB). Apoptosis is a mechanism used by infected cells to control the spread of pathogens. Interactions between the HIV Tat protein and AKT1 and RELA inhibit apoptosis, and lead to the survival and proliferation of cells [38], [39]. Activation of NF-κB in turn activates a number of survival genes. This strategy might help HIV to spread to other cells. The activation of survival genes might also inadvertently promote the growth and proliferation of cancer cells. Several cancer pathways highlighted in this work shared similar molecular machinery.

**Figure 5:**
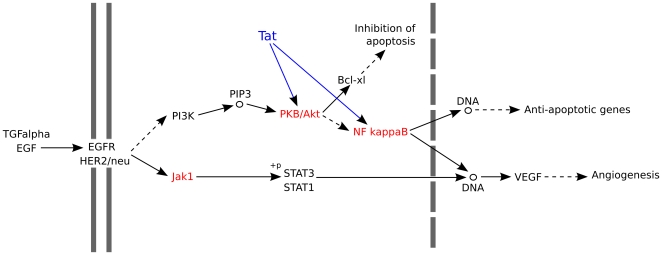
HIV host factors in the pancreatic cancer pathway. The pancreatic cancer pathway was reconstructed from Pancreatic Cancer Pathway (has:05212) in KEGG. Host factors are in red, including AKT1 (PKB/Akt), JAK1 (Jak1), and RELA (NF-κB). HIV protein Tat (in blue) interacted with AKT1 and NF-κB, activated the two proteins, and led to the expression of anti-apoptotic genes. The parallel solid lines represent the cell membrane. The dashed line represents the nuclear membrane.

The pancreatic cancer pathway was ranked first in the final ranking. There has been little data reported on the association between HIV and pancreatic cancer [30], [31], which might be due to the low prevalence of pancreatic cancer in the general population and its resulting difficulty of study. HIV host factors involved in the pancreatic cancer pathway (hsa05212) are highlighted (Figure 5). Many of these genes play important roles in a central pathway (the EGF/EGFR/JAK1/AKT/NF-κB axis) that might lead to the survival and proliferation of cancer cells, as noted above. Additionally, highly active anti-retroviral treatments (HAART) may also negatively affect the pancreas [40]. The cause of the increased incidence of pancreatic cancers in HIV/AIDS populations [30], [31] is not clear; it is speculated that the introduction of HAART significantly prolonged the life-span of HIV/AIDS patients, which might contribute to increases in tumor-associated deaths [31].

**Figure 6:**
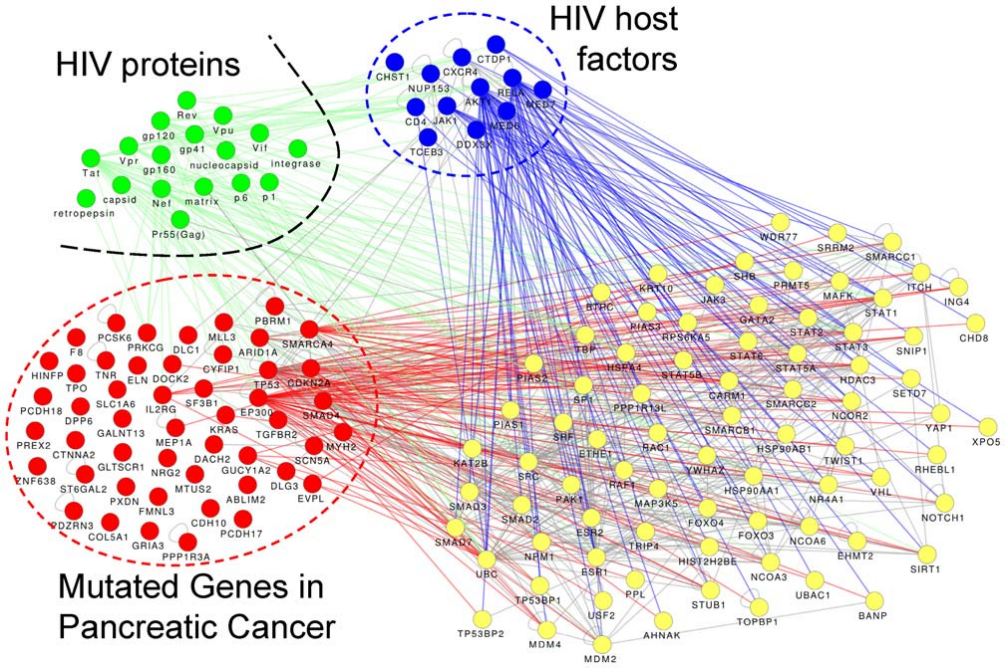
PPI network of HIV proteins, host factors, and genes that are mutated in pancreatic cancer. Connections between host factors (blue nodes) and mutated genes in pancreatic cancer (red nodes) were primarily relayed by other common interactors (‘hubs’, yellow nodes). HIV proteins (green nodes) interacted mostly with host factors and hubs.

To further elucidate the interactions between host factors and pancreatic cancers, 80 mutated genes implicated in pancreatic cancers were retrieved from a systematic screening survey [41]. A network of interactions among HIV proteins, host factors, and mutated genes in pancreatic cancers was constructed (Figure 6). The resulting network illustrated the fact that HIV host factors do not interact with mutated pancreatic genes directly; instead, a set of ‘proxies’ or ‘hubs’ are connected with both sets of genes. Interactions from the HIV-human interaction database revealed that HIV proteins share more interactions with host factors and these ‘hubs’, and fewer interactions with genes mutated in pancreatic cancer. At first glance, these results might suggest that the association between HIV infection and pancreatic cancer arises from the ‘common interaction partner’ method used in this work. However, in the four approaches used to study these data, the pancreatic cancer pathway ranked 1^st^, 6^th^, 8^th^, and 1^st^, respectively, and these associations were all statistically significant (Table 3). Thus, the association was not solely determined by indirect human protein-protein interactions. The existence of ‘proxy’ genes in the interaction network suggests that HIV infections and pancreatic mutations might lead to common outcomes, notably the activation of anti-apoptotic and pro-survival signaling pathways.

Chronic immune suppression was shown to increase the incidences of various cancers [25], [42]. HIV infection depletes CD4+ T-cells and macrophages, imposing a great impact on immune system functions. Recent studies revealed that CD4+ T-cells and macrophages are required in the clearance of senescent cells, which is critical to the prevention and regression of cancers [43]. Without functioning immune systems and these immune cells, senescent cells promote tumor growth and metastasis, though the underlying mechanism for this promotion remains to be elucidated [44].

Notably, several anti-retroviral agents were shown to have anti-tumor activities, and were used to treat various types of cancers [45]. Many HIV protease inhibitors also exhibited various degrees of kinase inhibition activity. For example, saquinavir, ritonavir, nelfinavir, and amprenavir were all able to inhibit phosphor-Akt (AKT1 was one of the host factors studied) and interfered with various signaling pathways. Among these protease inhibitors, nelfinavir has the most potent anti-cancer activity and was tested in clinical trials against pancreatic cancer [46]. Computational modeling and screening of human kinases revealed that nelfinavir inhibited multiple kinases, and its potent anti-tumor activity might come from this combined effect [47]. However, the tumor suppressor protein p21 (CDKN1A) was shown to confer HIV-1 resistance [48]. This and other studies suggest that anti-tumor drugs, specifically cyclin-dependent kinase (CDK) inhibitors, might serve as novel HIV/AIDS treatments [49], [50].

This work used a combined approach to identify associations between one specific pathogen (HIV) and human pathways. Various strategies are possible approaches to refining our method, such as comparisons of score combination and rank combination [51], and the use of a rank-score plot to identify the diversity of rankings and further improve combination results [52]. The identification of several cancer pathways associated with HIV was consistent with epidemiological reports of comorbidities and increased cancer risks in the HIV/AIDS population. The involvements of host factors in various cancer-related pathways also suggested the existence of common drugs or treatment options, as exemplified by HIV protease inhibitors and other anti-retroviral agents [45], and CDK inhibitors [49], [50]. Further investigations into the targets of anti-tumor drugs and their relationships with HIV host factors might reveal insights into novel treatment strategies for both HIV infection and cancers.

## Materials and Methods

### HIV Host Factors

HIV host factors were collected from the Human, HIV-1 Interaction Database [4] and several systemic screening studies. Overall, 1998 genes were identified and most (1431) were contributed by the HIV Interaction Database. Among these host factors, twelve (12) were reported by more than three studies and have been used as the set to be evaluated against the KEGG pathways.

### Human, HIV-1 Interaction Data and GO Annotation

Human, HIV-1 protein interactions were retrieved from the NCBI HIV-1, Human Protein Interaction Database [4]. Gene Ontology annotations of these human proteins were retrieved from the NCBI GeneRIF database (ftp://ftp.ncbi.nlm.nih.gov/gene/DATA/gene2go.gz). GO annotations have been assigned to GO terms one level below “Biological Process (GO:0008150)” using the “is_a” relationship in the Gene Ontology Database (revision: 1.2343, date: 24:10:2011). There were 24 terms in this level. For each term, the statistical significances of the proportional difference between the human genome and the set of HIV host factors were evaluated using a 2-sample proportion test.

### Human Protein-Protein Interactions

Human protein-protein interaction data were retrieved from the NCBI Interactions database (ftp://ftp.ncbi.nlm.nih.gov/gene/GeneRIF/, retrieved on Sep, 28, 2011). Eighty (80) genes mutated in pancreatic cancer were reported [41] and used to construct a protein-protein interaction network among HIV, host factors, and pancreatic cancer. None of these mutated genes overlapped with the 12 host factors. Protein-protein interaction networks were constructed and visualized using Cytoscape [53].

### KEGG Pathway Mapping

KEGG pathways and the genes that participate in these pathways were retrieved from the KEGG ftp site (ftp://ftp.genome.jp/pub/kegg/pathway/) [54]. Several files in the KEGG ftp site provide mapping between genes and pathways. Entrez Gene IDs of human targets were used to link HIV proteins to their respective KEGG pathways.

### Evaluation of HIV/KEGG Pathway associations

In this work, four approaches were applied to evaluate associations between HIV host factors and KEGG pathways. The rationales and details for applying these approaches are outlined here.

#### Common Genes

The first approach counts the number of genes appearing both in the set of HIV host factors and in individual pathways. If a pathway includes many HIV host factors, the association between the pathway and HIV would be highly significant. However, ranking pathways by the numbers of shared genes may be misleading. Large pathways with more genes may include more host factors by chance. Therefore, a bootstrap method was applied to estimate the distribution of shared gene numbers in random pathways, and to evaluate the statistical significance of the pathways. Pathways were ranked by their statistical significance (z-scores) and not by the numbers of common genes. The same procedure was applied to all four approaches. Details of the statistical testing procedures are described below.

#### Recovered Interactions

Host factors may contribute in different ways to virus-human interactions. Recovered interactions do not count the numbers of common genes, but do count the numbers of virus-human interactions. For example, two pathways with the same number of genes may both include three different host factors; the three host factors in pathway A may include eight human-virus interactions, and those in pathway B may only include five interactions. In this example, the association between HIV and pathway A would be stronger.

#### Co-expressed Genes

Some genes not in the host factor set may not have available human-virus interaction data. Co-expressions of these genes and host factors may provide another means by which to identify associations. Inference of gene associations through co-expressions has been widely adopted [55], [56]. Gene expression profiles from BioGPS [57] have been used to construct co-expressed relationships. For each gene, the expression levels across various tissue types have been used as the ‘expression profile’ of this particular gene. If more than one probe mapped to the same gene, the expression levels for these probes were averaged and assigned to the specific gene. Two genes were considered to be co-expressed if the Pearson correlation coefficient of their respective expression profiles across different tissue types was greater than 0.85.

#### Common Interaction Partners

The functions of proteins can be predicted using their connectivity information in protein-protein interaction networks [58], [59]. An association between two gene sets is considered to be strong if the two sets are connected by more common interaction partners between them. Common interaction partners of two genes are gene products that interact with both of the genes, excluding the two genes themselves (self-interacting homodimers). These common interaction partners were seen as ‘proxies’ or ‘bridges’ between two gene sets, and they represented indirect interactions between the two gene sets.

### Statistical Testing and Rank Combination

For each human KEGG pathway, 1,000 random pathways with the same numbers of genes were generated. The resulting distributions were used to evaluate the statistical significances of HIV-KEGG pathway associations. The means (*μ*) and standard deviations (*σ*) of the random distributions were calculated. The z-statistics of HIV host factors compared with these random pathways were evaluated. Therefore, *p*-values were estimated from the z-statistics.

Genes and gene products were ranked by their degrees of interaction in human protein-protein interaction networks and human-HIV protein interaction databases. When genes or gene products had the same degree, an equal and averaged rank was assigned. For example, if three genes with *N* interactions were placed in 7^th^, 8^th^, and 9^th^ places, then they each received an averaged rank of 8 ( = (7+8+9)/3).

KEGG Pathways were ranked by z-statistics calculated from the 4 measures outlined above: the number of overlapped genes, the number of HIV interactions, the number of co-expressed genes, and the number of common interaction partners in the human interactome. When applicable, rank combination was applied to merge ranks into a final rank. For example, Pathway A was ranked 2^nd^, 14^th^, 5^th^, and 7^th^ in 4 rankings, and Pathway B was ranked 8^th^, 1^st^, 33^rd^, and 2^nd^. After rank combination, their rank scores were 7 and 11, respectively. The rank of Pathway A therefore preceded that of Pathway B.

## Supporting Information

Table S1 **Rankings of KEGG Pathways by various approaches and rank combination.** Detailed information for the constructions of rankings by the four approaches and rank combination are included. For each approach, the means, standard deviations, z-statistics, *p*-values and ranks are provided. Ranks are based on z-statistics. The 220 KEGG pathways were sorted by combined ranks.
(XLS)

Table S2 **Enrichments and depletions of Gene Ontology biological processes.** Proportional differences in GO biological processes between the human genome and a set of HIV host factors were tested; z-statistics and *p*-values are provided. These GO processes were sorted by z-statistics. GO processes enriched in HIV host factors were placed at the top.
(XLS)
